# Hot stress: basal thermotolerance in Arabidopsis depends on two ethylene response factors, ERF95 and ERF97

**DOI:** 10.1093/plcell/koaa034

**Published:** 2020-12-18

**Authors:** P William Hughes

**Affiliations:** Department of Ecology, Environment, and Plant Sciences, Stockholm University, Svante Arrhenius väg 20, 114 18 Stockholm

Ethylene is a phytohormone implicated in many physiological and developmental pathways, and initiates a downstream signaling cascade by binding to ethylene-specific receptors such as ETR1 (ETHYLENE RESPONSE 1). The subsequent signaling cascade involves plant-specific transcription factors (TFs) such as ethylene response factors (ERFs), which regulate the expression of genes involved in specific functional responses, such as stimulating seed germination or resisting abiotic stress. To date, 65 ERFs have been identified, making them the largest subfamily of the well-known APETALA2/Ethylene Responsive Element Binding Protein (AP2/EREBP) family, whose members have diverse functions but share a characteristic DNA-binding domain (Müller and Munné-Bosch, 2015). Several ERFs have been well-characterized recently, including those involved in abiotic stress (see Xie et al., 2019). Although ethylene signaling is implicated in plant responses to heat stress, and ERF-mediated transcriptional regulation of downstream genes involved in thermotolerance is likely, the mechanism by which this is affected is currently unclear.

A recent paper by **Huang et al. (2021)** demonstrates that two ERFs (ERF95 and ERF97) are positive regulators of basal thermotolerance in *Arabidopsis thaliana*, thereby establishing that ethylene signaling is involved in heat stress response. In a previous paper, it was shown that ERF95 is directly regulated by *ETHYLENE INSENSITIVE 3* (*EIN3*), where it plays a role in salt stress tolerance (Zhang et al., 2011). Here, the authors demonstrate that ERF95 and ERF97, both of which act downstream of EIN3, regulate the expression of numerous plant heat stress genes in a heat-inducible manner.

The authors first examined basal thermotolerance in wild-type (WT) and mutant Arabidopsis lines, and demonstrated that ethylene signaling plays a positive role in plant basal thermotolerance. Seven-day-old Arabidopsis seedlings were immersed in a 43°C water bath for 22 min and their survival was monitored for a week after treatment. The survival rate of mutants lacking functional *EIN2* and *EIN3* alleles—two genes that act as positive regulators of ethylene signaling—was only 8%, which was markedly less than the survival rates of Col-0 WT plants (20%) or mutant lines constitutively overexpressing *EIN3* (80%). The authors also found that these mutants showed elevated levels of electrolyte leakage, an important measure of stress-induced injury and cell death. Next, the authors performed similar experiments using a transgenic line constitutively overexpressing *ERF95*, which showed a high survival rate (80%) and reduced electrolyte leakage relative to WT plants (see Figure). This result established a clear link between *ERF95* expression and enhanced basal thermotolerance.

**Figure:**
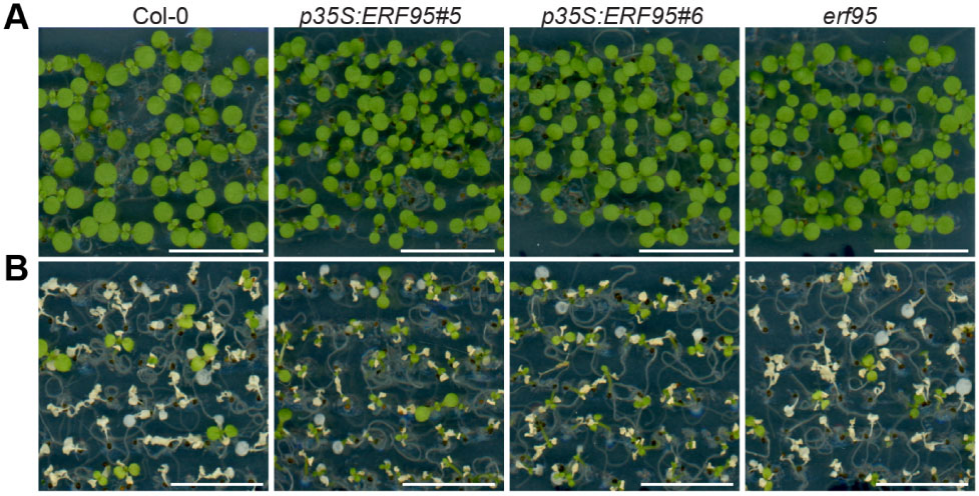
ERF95 confers higher tolerance to basal heat stress in Arabidopsis. Panels depict Arabidopsis seedlings from the Col-0 wild type and a transgenic line constitutively expressing ERF95. (A) Seedlings grown at 22°C under long-day conditions for 7 days. (B) Seedlings after exposure to a basal thermotolerance assay (i.e., immersion in a 43°C water bath for 22 min). *Adapted from*Huang et al. (2021), *Figure 2*.

Further experiments using immunoprecipitation assays showed that ERF95 coprecipitated with ERF97, indicating that these ERFs can form a heterodimer. Moreover, the authors also found that heat stress enhanced this interaction, suggesting that ERF95 and ERF97 may have a shared function as a heat-inducible protein complex involved in the plant heat stress response. Lines constitutively expressing ERF97 were then found to display a higher survival rate and less electrolyte leakage relative to WT plants. However, the authors also made a double mutant (*erf95,97*) that lacked functional alleles for ERF95 or ERF97, as well as a quadruple mutant (*erfq*) that lacked functional alleles for ERF95, ERF96, ERF97, and ERF98. The survival rate and amount of electrolyte leakage of *erf95,97* were similar to those of WT plants, while *erfq* plants showed a lower survival rate and higher electrolyte leakage than WT plants in response to basal heat treatment. Finally, the authors identified the downstream targets of ERF95 and ERF97 by performing mRNA sequencing on plants overexpressing these genes, some of which were subjected to the basal heat treatment. They found that while exposure to heat stress did not significantly induce the expression of either ERF, the two ERFs regulate overlapping sets of genes, many of which are known to be directly involved in heat stress response, such as *HSFA2*, an important heat shock TF.

In conclusion, the findings of Huang et al. (2021) establish that two ERFs, *ERF95* and *ERF97*, are implicated in basal thermotolerance in *Arabidopsis*. Moreover, they also showed that many heat-response genes were found to be regulated by both ERFs in a heat-inducible manner. These findings significantly improve our understanding of how ethylene signaling may be involved in thermal tolerance.
